# Pediatric Oral Commissure Burn

**DOI:** 10.5811/cpcem.2016.10.32247

**Published:** 2017-01-18

**Authors:** Kevin A. Hoffman, Christopher C. Trigger

**Affiliations:** * Michigan State University College of Osteopathic Medicine, Lakeland Health Emergency Medicine Residency Program, St. Joseph, Michigan; † Michigan State University College of Osteopathic Medicine and Lakeland Health, Department of Emergency Medicine, St. Joseph, Michigan

## INTRODUCTION

The labial, or oral, commissure is the site of a distinct pediatric injury that commonly presents as the result of an arc of electricity, which can preferentially injure the mucous membranes due to the surrounding electrolyte-rich saliva and the relatively low resistance of the tissues. The injury frequently involves a power cord of some type either being bitten or sucked on by the patient. The management of this injury can have profound impact on the aesthetic and functional outcomes for the patient.

## CASE REPORT

A five-year-old male presented to the emergency department at 2 a.m. after having bitten through a television power cable and suffering a burn injury to his mouth 20 minutes prior to arrival. The patient denied any other injuries and located pain only to the left side of his lips. He denied tongue pain.

Physical exam showed a gray and white eschar to the left oral commissure without any evidence of current or recent bleeding. Intraoral mucosa was only involved near the commissure and the tongue was uninjured. There was mild erythema and induration of the facial skin surrounding the eschar. The remainder of the exam was normal.

Acetaminophen was given for analgesia; however, no active intervention was required for the burn or resultant eschar. The patient was admitted to the general pediatric unit overnight for monitoring and pain management before being discharged the following day. He was referred to a regional burn specialist for non-emergent consultation within one week.

## DISCUSSION

Emergent management of an oral commissure burn depends on the extent of the injury. Evidence of airway involvement with singed nose hairs, soot-colored mucous, wheeze, stridor, voice changes or coughing may require definitive airway management with endotracheal intubation. General burn treatment principles such as the “rule of nines,” palmar surface area method, and Parkland formula, will guide treatment in those more extensively burned.

Oral commissure burns are classified by a system proposed by Al-Qattan et al which uses depth and location to guide treatment and prognosticate aesthetic and functional outcomes. In our case, the patient would be considered in the moderate category where splinting would be the recommended treatment of choice with the possibility of the need for commissuroplasty.^1^ Timing and technique of definitive operative management of oral commissure burns remains a controversial topic,^2^ making consultation with an expert in pediatric burn management crucial from the emergency physician’s perspective. Bleeding at the burn site is initially uncommon, though it may be seen between one and two weeks after the injury in up to 25% of cases. It is associated with exposure of the damaged labial artery with the slough of the eschar.^2^ Parents should be instructed on this point as well as to apply direct pressure to any bleeding.

## Figures and Tables

**Image:**
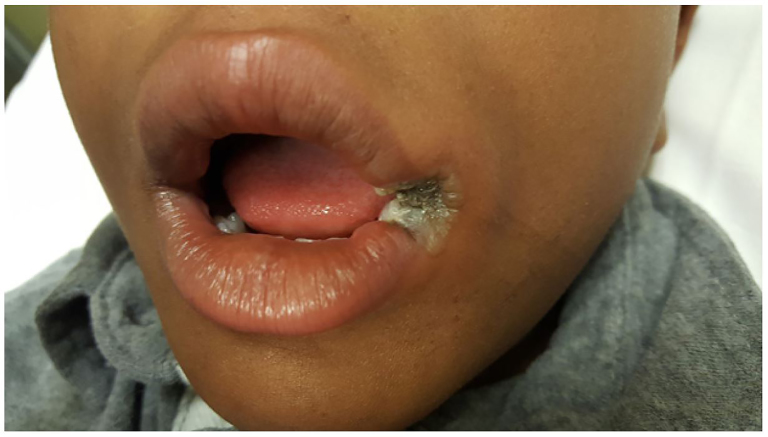
Left oral commissure burn with tissue destruction, eschar formation, and surrounding erythema in pediatric patient.
